# Detection of Vascular Notch3 Deposits in Unfixed Frozen Skin Biopsy Sample in CADASIL

**DOI:** 10.3389/fneur.2022.881528

**Published:** 2022-06-14

**Authors:** Akihiko Ueda, Makoto Nakajima, Yohei Misumi, Keiichi Nakahara, Satoru Shinriki, Masayoshi Tasaki, Hirotaka Matsui, Mitsuharu Ueda

**Affiliations:** ^1^Department of Neurology, Kumamoto University, Kumamoto, Japan; ^2^Department of Molecular Laboratory Medicine, Graduate School of Medical Sciences, Kumamoto University, Kumamoto, Japan; ^3^Department of Biomedical Laboratory Sciences, Graduate School of Health Sciences, Kumamoto University, Kumamoto, Japan

**Keywords:** CADASIL, Notch3 deposits, *NOTCH3* variants, skin biopsy, immunohistochemistry

## Abstract

This study aimed to evaluate the utility of immunohistochemical staining of vascular Notch3 deposits in biopsied unfixed frozen skin samples from patients with suspected cerebral autosomal dominant arteriopathy with subcortical infarcts and leukoencephalopathy (CADASIL). We analyzed vascular Notch3 deposits in unfixed frozen skin biopsy samples obtained from 43 patients with suspected CADASIL by immunohistochemistry using antibodies against the extracellular domain (ECD) of Notch3. We also sequenced the *NOTCH3* gene in all patients, as well as evaluated their symptoms and neuroimages. We found granular Notch3 ECD deposits in the vessel walls of unfixed frozen skin biopsy samples in 10 of the 43 suspected patients with CADASIL. All 10 cases with skin Notch3 ECD deposits also carried reported pathogenic variants in the *NOTCH3* gene associated with CADASIL. *NOTCH3* variants of unknown significance were found in the other four patients without vascular Notch3 ECD or granular osmiophilic material deposits in biopsied skin samples. The remaining 29 cases without vascular Notch3 ECD deposits did not have variants in the *NOTCH3* gene. Immunohistochemical evaluation of vascular Notch3 ECD deposits in unfixed frozen biopsied skin samples may be useful for detecting Notch3 deposits in CADASIL.

## Introduction

Cerebral autosomal dominant arteriopathy with subcortical infarcts and leukoencephalopathy (CADASIL) is a hereditary cerebrovascular disease caused by mutations in *NOTCH3* (1). Migraine, stroke recurrence, and cognitive decline are typical symptoms of CADASIL. Diffuse white matter lesions containing lesions in the temporal pole are characteristic findings of brain magnetic resonance imaging (MRI) in CADASIL. Genetic analysis of *NOTCH3* is required for the definitive diagnosis of CADASIL. Most patients with CADASIL have cysteine-related variants in *NOTCH3* that lead to an odd number of cysteine residues in epidermal growth factor-like repeats (EGFr) in the Notch3 extracellular domain (ECD). The occurrence of granular osmiophilic material (GOM) and Notch3 ECD deposits have been detected in brain vessels in CADASIL (2, 3).

For the diagnosis of CADASIL, simpler and less invasive detection of vascular Notch3 ECD deposits in the skin is a feasible alternative for detecting vascular pathogenic changes in the brain. Formalin-fixed paraffin-embedded (FFPE) sections, a standard method to fix biopsied samples, have previously been used for the immunohistochemical detection of vascular Notch3 deposits (4–7). However, immunohistochemical detection using FFPE samples has failed to detect Notch3 deposits due to structural alterations during tissue processing. In contrast, unfixed frozen tissue sections, in which the structural conformation of Notch3 ECD is considerably retained, maybe better for the detection of vascular Notch3 ECD deposits in patients with CADASIL (8).

In this retrospective case series, we evaluated the usefulness of immunohistochemical staining for the detection of vascular Notch3 ECD deposits using unfixed frozen skin biopsy samples obtained from 43 patients with suspected CADASIL.

## Materials and Methods

### Subjects

We consulted 380 patients who were suspected of developing CADASIL based on MRI T2 hyperintense lesions in periventricular white matter, deep white matter, and temporal pole white matter each attending doctor between 2008 and 2018 at Kumamoto University Hospital. We enrolled 43 suspected patients with CADASIL with informed consent, who agreed to participate in this study, for the investigation of the diagnostic utility of immunohistochemical detection of vascular Notch3 ECD deposits in the skin of these patients. We did not include patients with CADASIL reported in the previous study (8).

### Skin Biopsy Samples

We obtained 0.8 × 1.5 cm of skin biopsy samples from the upper arm of the 43 patients with suspected CADASIL. The biopsied skin samples were equally divided into three parts. The first part was fixed in 4% paraformaldehyde solution with 2.5% glutaraldehyde in 0.1-M sodium cacodylate buffer for electron microscopic analysis. The second part was rapidly frozen in isopentane and cooled in nitro liquid to prepare unfixed frozen sections for immunohistochemical staining of vascular Notch3 ECD deposits. The third part was fixed in 4% paraformaldehyde in phosphate buffer solution (PBS) for standard histopathological examinations.

### Immunohistochemical Staining of Vascular Notch3 ECD Deposits

For immunohistochemical staining of vascular Notch3 ECD deposits, we used 10-μm unfixed frozen skin sections. The sections were stained with rabbit antisera against Notch3 ECD (amino acid residues 1,555–1,569), which was prepared according to a previous study (9), overnight at 4°C. The sections were then washed with PBST for 3 h or more. To decrease the non-specific reaction of the primary antibodies, we prolonged the time of washing the sections in this step. The sections were then incubated with horseradish peroxidase (HRP)-conjugated goat secondary antibodies against rabbit immunoglobulin (Agilent, Santa Clara, CA, United States) for 2 h. Then, the sections were washed five times in phosphate-buffered saline with Tween20 (PBST). The sections were then incubated with 0.3 mg/ml diaminobezidine (Dojin Laboratories, Kumamoto, Japan), 0.65 mg/ml of sodium azide, and 100 μl of 30% hydrogen peroxide for 2 min, and counterstained with Victoria blue to visualize the internal elastic lamina.

### Electron Microscopy

Electron microscopy was performed as previously described (10). Briefly, the samples were post-fixed in buffered osmium tetroxide, dehydrated in ascending grades of ethanol, and embedded in Epon. Semi-thin sections were cut and stained with toluidine blue to select arteries of the appropriate size for thin sectioning. Thin sections were double-stained with uranyl acetate and lead citrate, and examined by transmission electron microscopy (TH 7700, HITACHI, Tokyo).

### Genetic Analysis and Clinical Presentations

We sequenced the *NOTCH3* gene in the 43 patients with suspected CADASIL using a next-generation sequencing panel as follows. We had designed a screening panel of genes for use with the Illumina TruSeq Custom Amplicon platform (Illumina, Inc., San Diego, CA, United States). The panel includes amplicons defining all coding exons of the 27 genes whose mutations are known to cause cerebral small vessel diseases including *NOTCH3*. Sequencing was performed using the MiSeq (Illumina, Inc.). The obtained sequences were aligned to the reference genome (GRCh37hg19) using MiSeq Reporter software (Illumina, Inc.). The generated virtual contact file (VCF) files containing variant calls were reviewed and further filtered. The clinical significance of the *NOTCH3* variants detected in the patients was assessed using ClinVar (https://www.ncbi.nlm.nih.gov/clinvar/). Pathogenicity of *NOTCH3* variants was predicted using PolyPhen-2 (http://genetics.bwh.harvard.edu/pph2/index.shtml) and MutationTaser2021 (https://www.genecascade.org/MutationTaster2021/#transcript). We analyzed the frequencies of each *NOTCH3* variant using the Human Genetic Variation Database (HGVD) (https://www.hgvd.genome.med.kyoto-u.ac.jp/about.html) for the Japanese population and the Genome Aggregation Database (gnomAD) (https://gnomad.broadinstitute.org/) for the general population. Symptoms and MRI findings using the Fazekas scale for white matter lesions were also evaluated.

## Results

### Vascular Notch3 ECD Deposits in Patients With and Without *NOTCH3* Mutations

**Figure 1:**
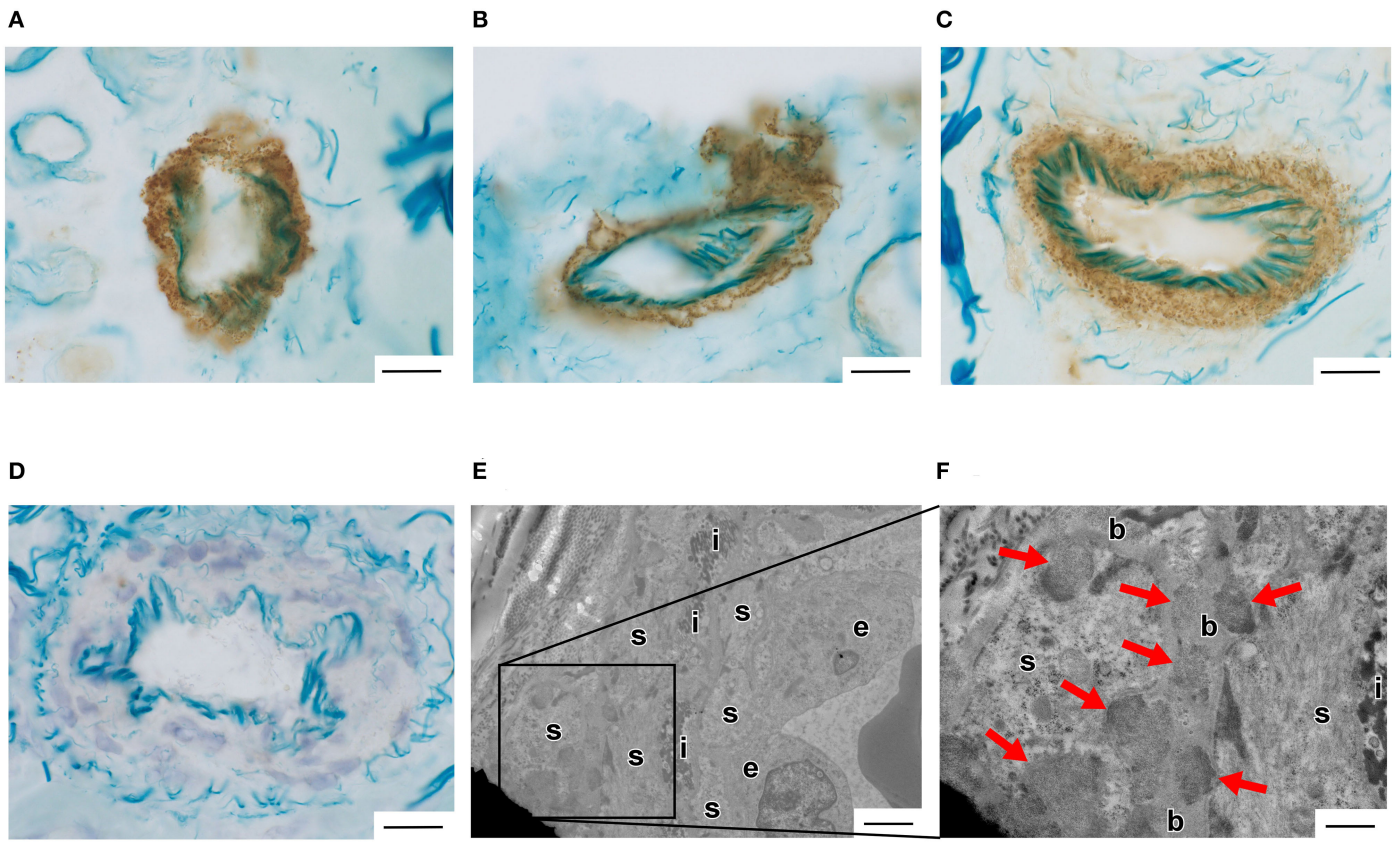
**(A–D)** Vascular Notch3 ECD deposits were detected by immunohistochemical staining using anti-Notch3 ECD antibodies in an unfixed frozen skin biopsy sample obtained from a patient with cerebral autosomal dominant arteriopathy with subcortical infarcts and leukoencephalopathy (CADASIL). **(A)** Case 2 with the pathogenic *NOTCH3* p.Arg110Cys variant. **(B)** Case 6 with the pathogenic *NOTCH3* p.Trp1003Cys variant. **(C)** Case 9 with the pathogenic *NOTCH3* p.Tyr1021Cys variant. **(D)** Case 35 with the likely benign variants of *NOTCH3* p.Arg75Gln (Table 1). Bars = 20 μm. **(E,F)** Vascular granular osmiophilic material (GOM) deposits were detected by electron microscopic analysis in a biopsied skin sample obtained from a patient with CADASIL (Case 9 with the pathogenic *NOTCH3* p.Tyr1021Cys variant, Table 1). Arrows indicate GOM deposits. e, endothelial cells; s, smooth muscle cells; i, internal elastic lamina; b, basal lamina. **(E)** Bar = 2 μm. **(F)** Bar = 400 nm.

Vascular Notch3 ECD deposits in unfixed frozen skin sections were visualized as granular dots in the arterial walls by immunohistochemical staining in 10 of the 43 patients with suspected CADASIL (Figures 1A–F, Table 1). Although amounts of granular Notch3 deposits were slightly different among patients with CADASIL, we found Notch3 deposits in all the arterioles. We detected Notch3 deposits in 8 (62%) of 13 patients with lesions in the temporal pole and 2 (7%) of 30 patients without lesions in the temporal pole (Table 2). We detected Notch3 deposits in 10 (34%) of 29 patients with Fazekas grade 3 white matter lesions and did not find them in 13 patients with Fazekas grade 1–2 white matter lesions (Table 2). GOM deposits were observed by electron microscopy (Figures 1E,F). The location and morphological features of the vascular Notch3 ECD and the GOM deposits were similar. We found Notch3 ECD deposits in all the three randomly selected CADASIL cases with GOM deposits (Table 1). Moreover, sequencing revealed that all 10 patients with vascular Notch3 ECD deposits had pathogenic or likely pathogenic variants of *NOTCH3*, such as p.Arg110Cys, p.Tyr258Cys, p Cys408Arg, p.Cys516Phe, p. Trp1003Cys, and p.Tyr1021Cys, which are reportedly associated with CADASIL (Table 1). *NOTCH3* p.Arg110Cys and p.Tyr258Cys variants are located in Notch3 EGFr domains 1–6 (case 2 and 10), and *NOTCH3* p.Cys408Arg, p.Cys516Phe, p.Trp1003Cys, and p.Tyr1021Cys variants are located in Notch3 EGFr domains 7–34 (cases 1 and 3–9). We did not find significant correlations between the degree of Notch3 deposits and *NOTCH3* mutation location. Based on the ClinVar database, the *NOTCH3* p.Arg75Gln variant is “likely benign.” The other five variants, such as p.Thr900Pro, p.Leu989Arg, p.Cys1372Gly, p Glu1373Gly, p.Ala1649Thr, and p.Gly1650Ser, were not found in the ClinVar database. According to PolyPhen-2 and MutationTaser2021, p.Leu989Arg, p.Ala1649Thr, and p.Gly1650Ser were predicted to be “benign” and p.Thr900Pro, p.Cys1372Gly, and p Glu1373Gly were predicted to be “probably damaging” and “deleterious” (Table 1).

**Table 1:** Skin vascular Notch3 ECD deposits and *NOTCH3* variants in 43 patients with suspected CADASIL.

**Case no**.	**Age**	**Sex**	**Skin vascular Notch3 ECD deposits**	**Skin vascular GOM deposits**	***NOTCH3* variants**	**EGFr domain no**.	**ClinVar**	**PolyPhen-2**	**MutationTaster2021**	**HGVD (allele frequency in the Japanese population)**	**gnomAD (allele frequency in the global population)**	**Fazekas grades**	**Lesions in the temporal pole**	**Clinical findings**	**Family history of stroke**
1	61	F	+	NA	p.Trp1003Cys	26	Likely pathogenic	Probably damaging	Deleterious	Unknown	Unknown	3	+	Stroke	+
2	62	F	+	NA	p.Arg110Cys	2	Pathogenic	Probably damaging	Deleterious	Unknown	0.000004	3	+	Stroke	+
3	51	F	+	+	p.Trp1003Cys	26	Likely pathogenic	Probably damaging	Deleterious	Unknown	Unknown	3	+	Dizziness, hemorrhage	+
4	41	M	+	+	p.Cys408Arg	10	NA	Probably damaging	Deleterious	Unknown	Unknown	3	+	Migraine	+
					p.Cys1410Tyr	NA (LNR1)	NA	Probably damaging	Deleterious	Unknown	Unknown				
5	45	M	+	NA	p.Trp1003Cys	26	Likely pathogenic	Probably damaging	Deleterious	Unknown	Unknown	3	+	Stroke	+
					p.Ala1649Thr	NA	NA	Benign	Benign	Unknown	Unknown				
6	48	M	+	NA	p.Trp1003Cys	26	Likely pathogenic	Probably damaging	Deleterious	Unknown	Unknown	3	+	Depression	+
7	42	M	+	NA	p.Trp1003Cys	26	Likely pathogenic	Probably damaging	Deleterious	Unknown	Unknown	3	+	Stroke	+
8	68	M	+	NA	p.Cys516Phe	13	Pathogenic	Probably damaging	Deleterious	Unknown	Unknown	3	−	Cognitive decline	−
9	50	M	+	+	p.Tyr1021Cys	26	Pathogenic	Probably damaging	Deleterious	Unknown	Unknown	3	+	Stroke	+
10	54	F	+	NA	p.Tyr258Cys	6	Pathogenic	Probably damaging	Deleterious	Unknown	Unknown	3	−	Stroke, cognitive decline	−
11	51	M	−	−	−	NA	NA	NA	NA	NA	NA	3	−	Stroke, cognitive decline	+
12	47	M	−	−	p.Leu989Arg	25	NA	Benign	Benign	Unknown (0.00030 in 8.3KJPN*)	Unknown	3	−	Cognitive decline	−
13	54	M	−	−	−	NA	NA	NA	NA	NA	NA	3	−	Headache, Stroke, cognitive decline	+
14	46	M	−	−	−	NA	NA	NA	NA	NA	NA	3	−	Cognitive decline	−
15	43	M	−	NA	−	NA	NA	NA	NA	NA	NA	1	−	Cerebral hemorrhage	−
16	49	M	−	NA	−	NA	NA	NA	NA	NA	NA	3	+	Cognitive decline	−
17	44	F	−	−	−	NA	NA	NA	NA	NA	NA	3	+	Headache, stroke, cognitive decline	+
18	58	F	−	NA	−	NA	NA	NA	NA	NA	NA	3	−	Cognitive decline	+
19	57	M	−	NA	−	NA	NA	NA	NA	NA	NA	3	−	Stroke	+
20	66	F	−	NA	−	NA	NA	NA	NA	NA	NA	3	+	Headache	+
21	48	M	−	NA	−	NA	NA	NA	NA	NA	NA	2	−	Headache, stroke, MCI	+
22	56	F	−	NA	−	NA	NA	NA	NA	NA	NA	2	−	Mood disorder	+
23	49	M	−	NA	−	NA	NA	NA	NA	NA	NA	3	−	Cognitive decline	−
24	55	M	−	NA	−	NA	NA	NA	NA	NA	NA	3	+	Asymptomatic stroke	−
25	56	F	−	−	p.Cys1372Gly	34	NA	Probably damaging	Deleterious	Unknown	Unknown	2	−	Migraine	−
					p.Gly1650Ser	NA	NA	Benign	Benign	Unknown	Unknown				
26	55	F	−	−	p.Cys1372Gly	34	NA	Probably damaging	Deleterious	Unknown	Unknown	2	−	Cognitive decline	−
					p.Thr900Pro	23	NA	Probably damaging	Deleterious	Unknown	Unknown (0.000004in TOPMed**)				
					p Glu1373Gly	34	NA	Probably damaging	Deleterious	Unknown	Unknown				
27	35	M	−	NA	−	NA	NA	NA	NA	NA	NA	3	−	Migraine, stroke	+
28	55	F	−	−	−	NA	NA	NA	NA	NA	NA	3	−	Asymptomatic aneurism	+
29	62	M	−	−	−	NA	NA	NA	NA	NA	NA	3	−	Brain hemorrhage	−
30	60	M	−	NA	−	NA	NA	NA	NA	NA	NA	3	−	Stroke	+
31	67	M	−	−	−	NA	NA	NA	NA	NA	NA	3	−	Stroke, Cognitive decline	+
32	67	F	−	−	−	NA	NA	NA	NA	NA	NA	3	−	Cognitive decline	−
33	49	F	−	−	−	NA	NA	NA	NA	NA	NA	2	+	MCI	−
34	61	M	−	−	−	NA	NA	NA	NA	NA	NA	3	−	Stroke, cognitive decline	+
35	38	F	−	−	p.Arg75Gln	1	Likely benign	Probably damaging	Benign	Unknown	Unknown	2	−	Migraine	−
36	42	F	−	NA	−	NA	NA	NA	NA	NA	NA	1	−	Dizziness	−
37	31	M	−	NA	−	NA	NA	NA	NA	NA	NA	NA	−	Headache	+
38	45	F	−	−	−	NA	NA	NA	NA	NA	NA	2	−	Stroke	+
39	50	F	−	−	−	NA	NA	NA	NA	NA	NA	2	−	MCI, mood disorder	+
40	53	F	−	−	−	NA	NA	NA	NA	NA	NA	2	−	Migraine	−
41	53	F	−	−	−	NA	NA	NA	NA	NA	NA	3	−	Stroke	+
42	56	M	−	−	−	NA	NA	NA	NA	NA	NA	2	−	MCI	−
43	63	M	−		−	NA	NA	NA	NA	NA	NA	2	−	Asymptomatic	−

*EGFr, epidermal growth factor like repeats; gnomAD, the Genome Aggregation Database; GOM, granular osmiophilic material; HGVD, the Human Genetic Variation Database; MCI, mild cognitive impairment; NA, not available*.

^*^Tadaka et al., 3.5KJPNv2: an allele frequency panel of 3,552 Japanese individuals including the X chromosome. **Hum Genome Var** (2019) 6:28.

^**^ TOPMed (“https://topmed.nhlbi.nih.gov/”).

**Table 2:** Number of patients with and without skin Notch3 deposits among patients with suspected CADASIL.

	**Total number**	**Skin Notch3 positive**	**Skin Notch3 negative**
Patients with pathogenic *NOTCH3* variants associated with CADASIL	10	10 (100%)	0 (0%)
Patients with *NOTCH3* variants of unknown significance	4	0 (0%)	4 (100%)
Patients without *NOTCH3* variants	29	0 (0%)	29 (100%)
Patients with lesions in the temporal pole	13	8 (62%)	5 (38%)
Patients without lesions in the temporal pole	30	2 (7%)	28 (93%)
Patients with Fazekas grade 3 white matter lesions	29	10 (34%)	19 (66%)
Patients with Fazekas grade 1–2 white matter lesions	13	0 (0%)	13 (100%)

Four of the 33 patients without vascular Notch3 ECD or GOM deposits in skin biopsy samples (cases 12, 25, 26, and 35) (Table 1) had *NOTCH3* variants of unknown significance, such as p.Arg75Gln, p.Thr900Pro, p.Leu989Arg, p.Cys1372Gly, p Glu1373Gly, and p.Gly1650Ser. The remaining 29 patients without vascular Notch3 ECD deposits did not have any *NOTCH3* variants. We also investigated vascular GOM deposits in 16 of the 29 patients and found no GOM deposits in any of the 16 patients without vascular Notch3 ECD deposits or *NOTCH3* variants (Table 1).

### Brain MRI Findings in Patients With and Without Vascular Notch3 ECD Deposits

**Figure 2:**
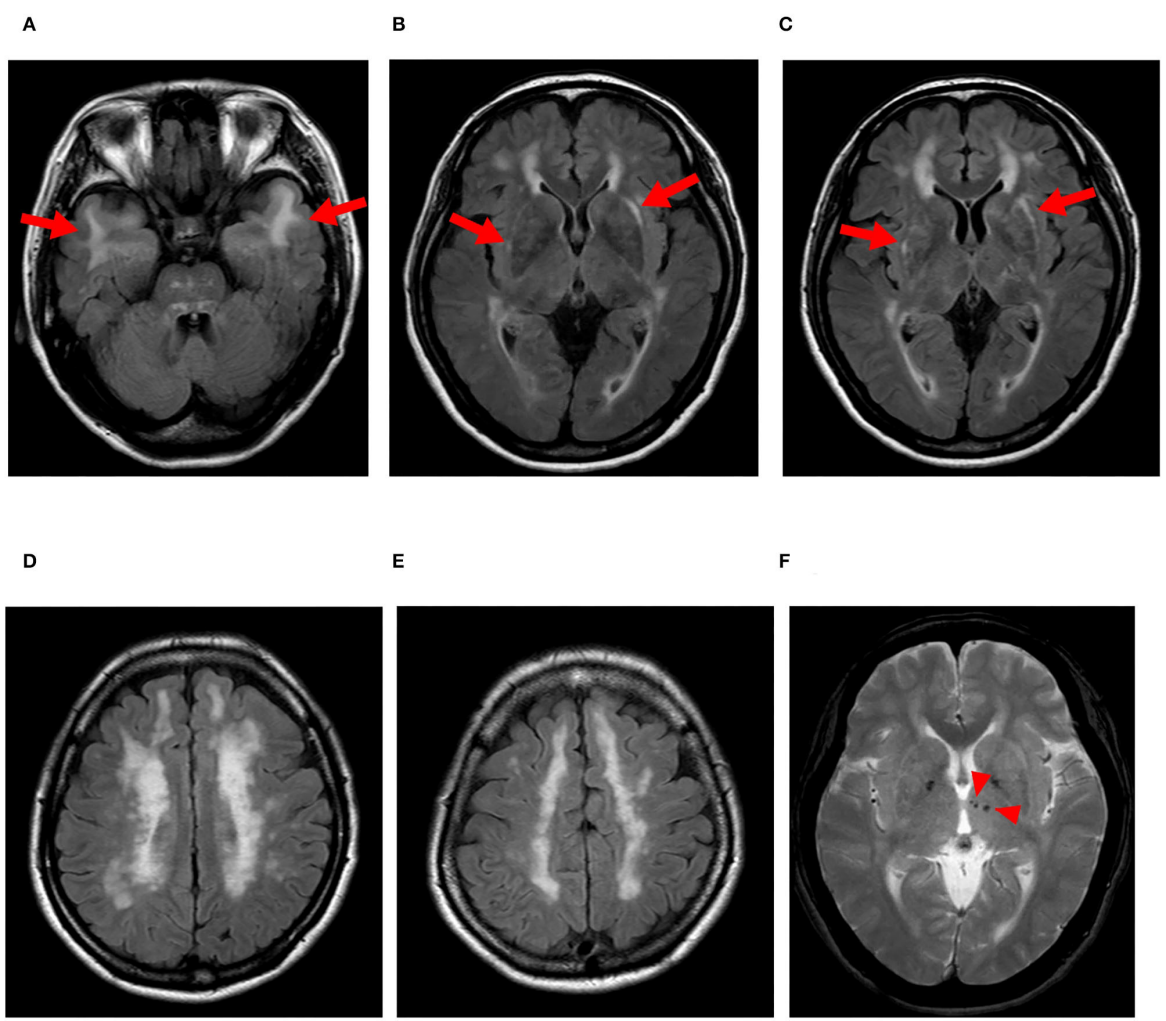
Representative MRI findings of CADASIL. Case 2 with the pathogenic *NOTCH3* p.Arg110Cys variant. **(A–E)** FLAIR images, **(F)** T2-star weighted image. **(A)** Arrows indicate lesions in the temporal pole. **(B,C)** Arrows indicate lesions in the extra capsule of the putamen, and **(F)** arrowheads indicate microbleeds.

We found lesions in the temporal pole on MRI (Figure 2) and a family history of stroke in eight of the 10 (80%) patients with vascular Notch3 ECD deposits. Two patients (cases 8 and 10) with vascular Notch3 ECD deposits had neither lesion in the temporal pole on MRI nor a family history of stroke (Table 1). In contrast, 5 of the 33 (15%) patients without vascular Notch3 ECD deposits (cases 16, 17, 20, 24, and 33) had lesions in the temporal pole. No *NOTCH3* variants were found in these five patients (Table 1).

## Discussion

In this case series study of 43 patients with suspected CADASIL, we detected vascular Notch3 ECD deposits in all 10 patients by immunohistochemical staining using unfixed frozen biopsied samples, which were confirmed to have pathogenic *NOTCH3* variants causing CADASIL. In contrast, conventional immunohistochemical staining using FFPE tissue samples fails to detect vascular Notch3 deposits in 5–15% of patients with CADASIL with pathogenic variants in *NOTCH3* (4–6). Therefore, unfixed frozen biopsied tissue samples may be more suitable than FFPE-biopsied tissue samples for the detection of vascular Notch3 ECD deposits in the skin.

Patients with CADASIL carrying pathogenic *NOTCH3* variants, which were mostly associated with cysteine replacement (11), located in the EGFr domains 7–34, reportedly showed milder phenotypes than those with *NOTCH3* variants located in the EGFr domains 1–6 (12). In addition, Gravesteijn et al. (13) recently reported that the amount of vascular Notch3 ECD and GOM deposits in the skin in patients with CADASIL with *NOTCH3* variants in EGFr 7–34 was lesser than that in those with *NOTCH3* variants in EGFr 1–6. In this case series study, we successfully detected vascular Notch3 ECD deposits in patients with CADASIL with both milder *NOTCH3* EGFr 7–34 variants and typical severe *NOTCH3* EGFr 1–6 variants by immunohistochemical staining using unfixed frozen biopsied skin samples (Table 1). Therefore, immunohistochemical staining using unfixed frozen biopsied skin samples seems to be suitable for detecting Notch3 deposits in CADASIL regardless of the amount of Notch3 ECD deposits.

Detecting Notch3 and GOM deposits are thought to be helpful for the diagnosis of CADASIL. Brain MRI findings reportedly varied considerably between patients with CADASIL and were dependent on the *NOTCH3* genotype (14). While the involvement of the anterior temporal pole and external capsule may be helpful for the diagnosis of CADASIL, these MRI findings were reportedly not sufficient for accurate diagnosis of CADASIL (14). Skin biopsy is less invasive than brain biopsy to directly confirm pathogenic Notch3 and GOM deposits in patients with suspected CADASIL. In this study, 10 of 43 cases were identified as positive of staining of Notch3 and/or GOM. We believe that skin biopsy is useful especially for detecting Notch3 deposits in patients with CADASIL with *NOTCH3* variants of unknown significance, while skin biopsy may not be essential for the diagnosis of patients with CADASIL with typical *NOTCH3* variants and typical MRI findings in the daily clinical practice.

This study is limited in that it had a small sample size. Large-scale studies including more patients with CADASIL with other genotypes are needed to determine the sensitivity and specificity of this immunohistochemical method in differentiating between CADASIL and other cerebral small vessel diseases.

## Conclusion

Immunohistochemical staining of vascular Notch3 ECD deposits in unfixed frozen skin sections may be useful over conventional immunohistochemical staining for detecting Notch3 deposits in CADASIL.

## Data Availability Statement

The raw data supporting the conclusions of this article will be made available by the authors, without undue reservation.

## Ethics Statement

This study was approved by the Human genome Ethics Committee of Kumamoto University. The patients/participants provided their written informed consent to participate in this study.

## Author Contributions

AU drafted the manuscript, devised the study concept and design, collected data, and performed the pathological examinations. SS and HM performed the gene analysis. MN, KN, MT, and YM revised the manuscript. MU revised the manuscript, devised the study concept and design, and supervised the study. All authors have contributed to the manuscript and approved the submitted version.

## Funding

This study was funded by a grant-in-aid for research on intractable diseases from the Japanese Ministry of Health, Labor, and Welfare (Grant No. 21FC1007).

## Conflict of Interest

The authors declare that the research was conducted in the absence of any commercial or financial relationships that could be construed as a potential conflict of interest.

## Publisher's Note

All claims expressed in this article are solely those of the authors and do not necessarily represent those of their affiliated organizations, or those of the publisher, the editors and the reviewers. Any product that may be evaluated in this article, or claim that may be made by its manufacturer, is not guaranteed or endorsed by the publisher.

## Abbreviations

CADASIL: cerebral autosomal dominant arteriopathy with subcortical infarcts and leukoencephalopathy
EGFr: epidermal growth factor-like repeats
ECD: extracellular domain
GOM: granular osmiophilic material
FFPE: formalin-fixed paraffin-embedded sections
PBS: phosphate buffer solution
HRP: horseradish peroxidase
DAB: diaminobenzidine.
